# Volunteer-led physical activity interventions to improve health outcomes for community-dwelling older people: a systematic review

**DOI:** 10.1007/s40520-020-01556-6

**Published:** 2020-04-30

**Authors:** S. E. R. Lim, N. J. Cox, Q. Y. Tan, K. Ibrahim, H. C. Roberts

**Affiliations:** 1 Academic Geriatric Medicine, University of Southampton, Southampton, UK; 2 NIHR ARC Wessex, University of Southampton, Southampton, UK; 3 NIHR Southampton Biomedical Research Centre, University of Southampton and University Hospital Southampton NHS Trust, Southampton, UK

**Keywords:** Volunteer, Physical activity, Exercise, Older adults, Health outcomes

## Abstract

**Background:**

Physical activity (PA) is important for older people to maintain functional independence and healthy ageing. There is strong evidence to support the benefits of physical activity interventions on the health outcomes of older adults. Nonetheless, innovative approaches are needed to ensure that these interventions are practical and sustainable.

**Aim:**

This systematic review explores the effectiveness of volunteer-led PA interventions in improving health outcomes for community-dwelling older people.

**Methods:**

Five databases (MEDLINE, Embase, CINAHL, PEDro, Cochrane library) were systematically searched for studies using trained volunteers to deliver PA interventions for community-dwelling older people aged ≥ 65 years. Meta-analysis was not conducted due to included study heterogeneity.

**Results:**

Twelve papers describing eight studies (five papers reported different outcomes from the same study) were included in the review. All eight studies included strength and balance exercises and frequency of PA ranged from weekly to three times a week. Volunteer-led exercises led to improvements in functional status measured using the short physical performance battery, timed up and go test, Barthel Index, single leg stand, step touch test, chair stand test, and functional reach. Frailty status identified by grip strength measurement or the use of long-term care insurance improved with volunteer-led exercises. Interventions led to improvement in fear of falls and maintained or improved the quality of life. The impact on PA levels were mixed.

**Conclusion:**

Limited evidence suggests that volunteer-led PA interventions that include resistance exercise training, can improve outcomes of community-dwelling older adults including functional status, frailty status, and reduction in fear of falls. More high-quality RCTs are needed to investigate the effects of volunteer-led PA interventions among older people.

## Background

Physical activity (PA) is important for a healthy ageing and the quality of life of older people. The maintenance of physical activity enables older people to preserve and maintain their ability to carry out activities of daily living (ADL), which is fundamental for their independence and quality of life. Among community-dwelling older adults, physical activity interventions have been shown to improve physical activity levels [1] and functional outcomes [2]. In both reviews, the interventions in all included studies were delivered by healthcare professionals or exercise specialists and conducted among older adults aged 50 years and above.

National guidelines have been published to raise awareness regarding the importance of physical activity [3, 4]. The current recommended physical activity level for adults aged 65 years and older is 150 min a week of moderate intensity activity plus muscle strengthening exercises on two days [5]. This is often expressed as 30 min of brisk walking or equivalent activity of five days a week, although 75 min of vigorous intensity activity spread across the week or a combination of moderate and vigorous activity are sometimes suggested [6]. However, studies have shown that many older adults do not meet the recommended guidance. In a UK study, 238 community dwelling older people (age ≥ 65 years) from a single general practice wore an accelerometer to study their average daily step-counts and time spent at different physical activity levels [7]. Only 2.5% of participants achieved the recommended 150 min weekly of moderate intensity activity in bouts of 10 min or more. In the US, it is reported that only 11% of older adults meet the national recommendations of physical activity levels [8]. These findings demonstrate the high prevalence of physical inactivity among community-dwelling older adults.

Physical inactivity has been acknowledged as a global health issue and is estimated to contribute to 6% of global deaths [9]. In the UK, the cost of physical inactivity to the NHS in 2013 was estimated to be £0.9 billion [10]. Effective interventions are needed to promote increased physical activity among older people to reduce their risk of adverse health outcomes such as coronary heart disease, type 2 diabetes, increased frailty and pre-mature mortality [11, 12].

Increasingly, volunteers are recognised to play an important role in improving patient experience and care [13]. In some care settings, volunteers are being seen as an integral part of the care team rather than as an ‘add-on’ [14]. Given the impact of physical inactivity on the health outcomes of older adults and over stretched health and social care staff, innovative ideas are needed to ensure that older adults are encouraged to lead an active and healthy lifestyle. There are cost implications in having an intervention delivered by an exercise specialist or healthcare professional. In a US study which evaluated the implementation of a physical activity promotion programme for older adults (age > 60 years) in four locations, one site did not hire an exercise specialist as it was felt that the cost was unsustainable [15]. Instead trained volunteers were used to deliver the interventions and it was reported that the volunteers were able to help increase uptake and adherence to exercise. As the use of trained volunteers is more cost-effective in comparison to professionals or specialists, these interventions are likely to be more sustainable. Other benefits that volunteers may bring include breaking down communication barriers and influencing behaviour through positive role modelling [16]. This review aims to explore the evidence on the use of trained volunteers to deliver physical activity interventions for community-dwelling older adults and its impact on health outcomes.

## Methods

### Search strategy and study selection

**Fig. 1:**
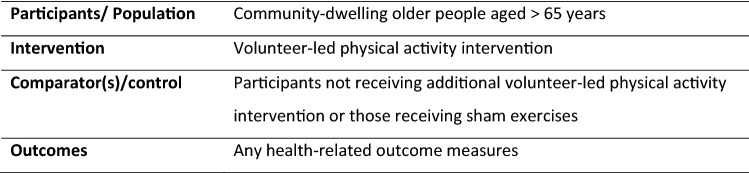
PICOs statement

A systematic search of the literature was conducted on five electronic databases: MEDLINE, Embase, CINAHL, PEDro, and Cochrane library, from inception to May 2019. The inclusion criteria were as follows: (1) a volunteer-led physical activity intervention, (2) in community-dwelling older people aged ≥ 65 years, (3) which included any health-related outcome measures (Fig. 1). The outcome measures presented in this review included physical activity levels, functional status, frailty status, fear of falls and quality of life. The search strategy was developed by two authors (SL and HR). All study designs were included in the review. Studies which were conducted in acute hospital settings or written in languages other than English, were excluded. This review was registered on PROSPERO: CRD42020154607.

The screening of titles and abstracts were conducted by two reviewers independently (SL and HR). Articles that were selected by at least one reviewer were included for full text review. Two reviewers (SL and NC) independently reviewed the full texts and identified relevant studies for final analysis. Any disagreements were resolved through discussion.

### Data extraction and analysis

A pre-defined data extraction template was developed by the authors and data extraction was conducted by two reviewers (SL and NC). The following data were extracted: author, country, study design, sample characteristics, outcome measures, description of the physical activity intervention, and main study findings. Important details regarding the volunteer-led physical activity intervention included the type of intervention (e.g.: mobility, strength, balance or aerobic exercises), where they were delivered, frequency and duration of the intervention and follow-up period. Due to the heterogeneity of interventions delivered and outcome measures used, statistical pooling of data was not conducted. PRISMA guidance was adhered to in the reporting of this review [17].

### Quality rating

The quality of studies was assessed by two reviewers (KI and QT) using the Joanna Briggs Institute’s critical appraisal tools [18]. The tools applied were selected based on study design. Randomised controlled trials were given a score out of 13 and non-randomised experimental studies were given a score out of 9. Any disagreements were resolved by consensus or through third party consultation.

## Results

**Fig. 2:**
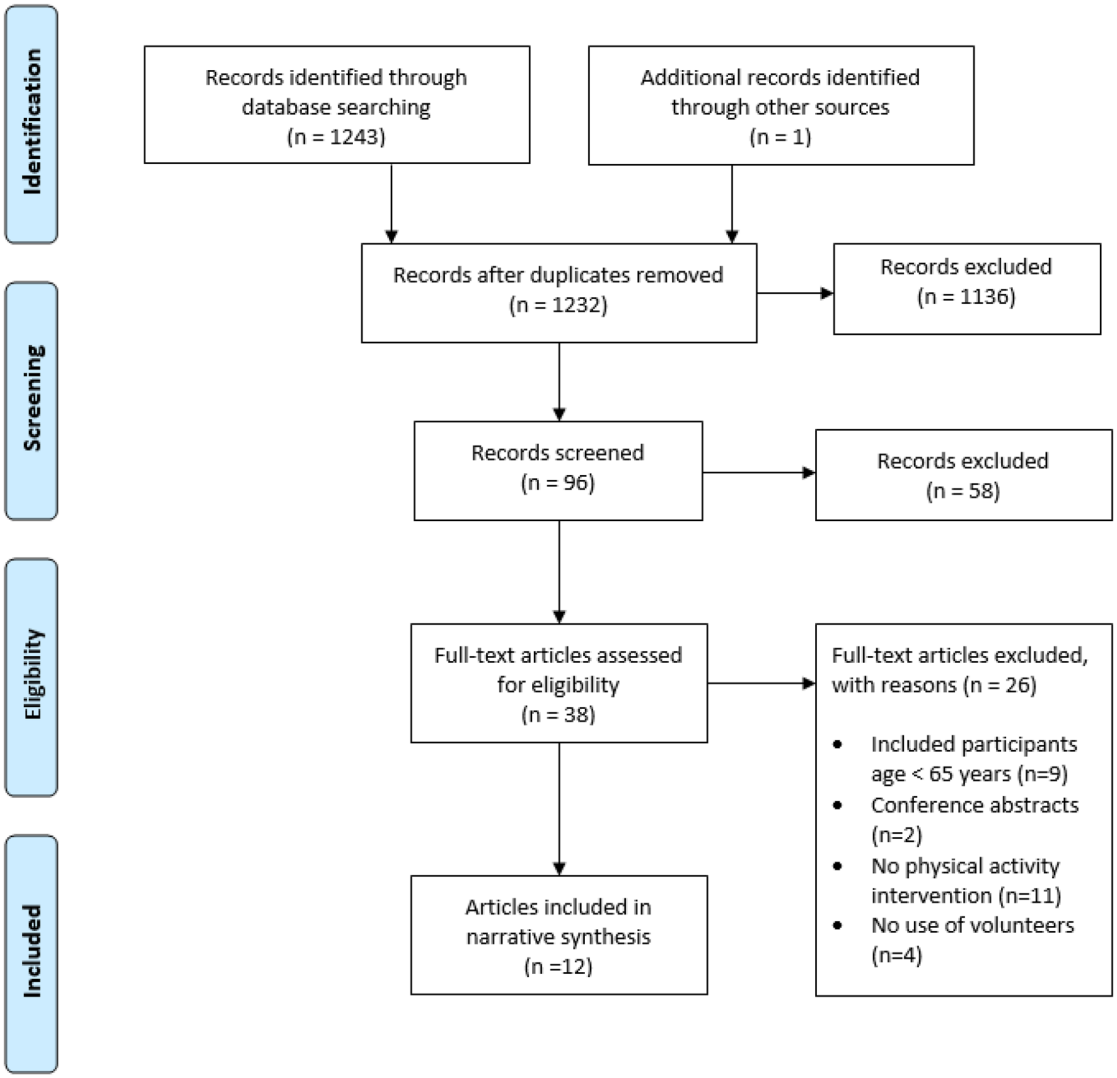
Study selection flowchart according to PRISMA checklist

The initial search identified 1232 articles after duplicates were removed (Fig. 2). 97 articles were selected for abstract review and 37 articles were selected for full text review. Five systematic review articles were identified in the abstract screening stage and articles included in those reviews were screened, adding one additional article to the final full text review of 38 articles. 12 papers met the inclusion criteria and were selected for further analysis (Table 1). The quality of the studies is presented in Table 1.

**Table 1:** Characteristics of included studies

Study, Country	Study design	Sample	Intervention	Follow-up	Main findings	Study quality
Five articles publishing data from the same study						
Haider 2017, Austria [19]	RCT	*N* = 80, mean age 82 years community-dwelling older adults	Twice weekly mobility and strength exercises, plus nutritional intervention	12 weeks	OR of 2.7 (95% CI 1.0–7.2) in improvement of handgrip by 2 kg among frail participants, compared to robust or pre-frail participants	9/13
Kapan 2017, Austria [20]	RCT	*N* = 80, mean age 82 years community-dwelling older adults	Twice weekly mobility and strength exercises, plus nutritional intervention	12 weeks	Reduction in fear of falling (FES-I score) from 44.1 to 29.9 vs control 41.4–41.5 (*p* = 0.016)	9/13
Kapan 2017, Austria [21]	RCT	*N* = 80, mean age 82 years community-dwelling older adults	Twice weekly mobility and strength exercises, plus nutritional intervention	12 weeks	No significant difference in QoL measured by WHOQOL-BREF and WHOQOL-OLD	9/13
Luger 2016, Austria [22]	RCT	*N* = 80, mean age 82 years community-dwelling older adults	Twice weekly mobility and strength exercises, plus nutritional intervention	12 weeks	MNA-LF (mean change 1.54 (0.51–2.56) and SHARE-FI (mean change − 0.71 (− 1.07 to − 0.35) within subjects. No statistically significant changes between subjects	9/13
Winzer 2019, Austria [23]	RCT	*N* = 80, mean age 82 years community-dwelling older adults	Twice weekly mobility and strength exercises, plus nutritional intervention	12 weeks	Improvement in overall activity levels 0.69 h/day (95% CI 0.21–1.18)	7/13
Chen 2016, Taiwan [24]	Cluster RCT	*N* = 127, mean age 79 years, care home residents	Three times a week resistance exercises in care home	6 and 12 months	Improvement in grip strength 3.1 kg (95% CI 1.86–4.4) at 12 months	9/13
Iliffe 2014, UK [25]	Cluster RCT	*N* = 1256, age ≥ 65 years community-dwelling older adults	Weekly community group exercise and home exercises (FaME vs OEP vs control)	12 months	Proportion of participants reported reaching the recommended PA target of 150 min of MVPA increased to 43% from 41%	9/13
Nanduri 2018, USA [26]	Quasi-experimental	*N* = 40, mean age 80 years, older adults in assisted living facility and home-dwelling older adults)	Weekly exercise including posture, strength, flexibility and balance, plus education classes	6 months	Improvement in TUG by 2.3 s (*p* < 0.001), functional reach 4.4 cm (*p* < 0.001) among home-dwelling older adults	6/9
Waters 2011, New Zealand [27]	Quasi-experimental, 3 arm	*N* = 118, mean age 75 years, community-dwelling older adults	Weekly group exercises adapted from Otago Exercise Program (Peer-led vs Age Concern Otago vs control)	10 weeks	Similar improvement in functional outcomes (TUG, 30 s chair stand) in peer-led and Age Concern Otago group at 12 months	8/9
Yamada 2017, Japan [28]	Prospective cohort study	*N* = 3240, mean age 77, community-dwelling older adults	Once or twice every two weekly groups exercise including aerobic, strength, flexibility and balance	4 years	Reduction in new long-term care insurance certification. HR 0.73 (95% CI 0.62–0.86) at 4 year follow up	8/9
Izutsu 2017, Japan [29]	Observational study	*N* = 127, mean age 76, community-dwelling older women	Weekly strength, balance and mobility exercises. Delivered by volunteer compared to healthcare professional	6 months	Both volunteers and health professionals had a maintaining or improving SF-36 scores in > 70% of participants	6/9
Healy 2008, USA [30]	Single-group, repeated measure	*N* = 335, mean age, 78.7, community-dwelling older adults	Twice weekly strength and balance exercise, plus cognitive-behavioural program	4 weeks	Improvement in FES at 6 months (*p* < 0.001) and 12 months (*p* = 0.001)	8/9

*RCT* Randomised controlled trial, *N* sample size, *CI* 95% confidence interval, *kg* kilogram, *FES* Falls Efficacy Scale, *FaME* falls management exercise, *OEP* Otago Exercise programme, *PA* physical activity, *MVPA* moderate-vigorous physical activity, *SF-36* Short Form 36 Questionnaire, *WHOQOL-BREF* WHO Quality of Life-BREF Questionnaire, *WHOQOL-OLD* WHO Quality of life Instrument-Older Adults Module, *MNA-LF* Mini nutritional assessment-long form, *SHARE-FI* The Survey of Health, ageing and Retirement in Europe-Frailty Instrument, *TUG* timed up and go test

### Study samples

12 papers were accepted after full text review. However, five papers published findings from the same study which explored the impact of a home-based physical and nutritional intervention on various outcome measures [19–23]. Eight studies were included in the narrative analysis, which included three randomised controlled trials [22, 24, 25], two quasi-experimental studies [26, 27], a large prospective cohort study [28], an observational study [29] and a single group repeated measure design study [30]. Six studies were conducted among older people who were living at home, one study included older people in assisted living facilities [26] and one study included nursing home residents [24]. The median sample size was 122 (IQR 65–796), and varied considerably from 40 [26] to 3240 [28] participants. The participant mean age in these studies ranged from 75.5 years [27] to 82.8 years [22].

### Interventions

Four studies provided brief descriptions on volunteer recruitment. Three studies [19, 29, 30] recruited volunteers from the community and one study [24] recruited from among those working in the nursing home where the intervention was conducted. Only three studies provided minimal description of volunteer characteristics. One RCT included only volunteers aged 50 years and over [19]. Another study [24] included mostly middle-age women as volunteers, and one [29] reported the mean age of the volunteers as 72 years old (*n* = 30). Volunteer training was reported in six studies; training duration ranged from 9 h [24] to 2 days [30]. The trainers included physiotherapists [28], physical therapists [29] and public health nurses [28, 29]. The role or professional background of the trainers were not clearly identified in four studies [19, 24, 27, 30]. Only one study reported on volunteer retention. The study by Iliffe et al. reported that 209 people expressed interest in volunteering, 71 received training and only 38 delivered the intervention [25].

The volunteer-led interventions delivered were heterogeneous, with four studies delivering exercise only interventions which all included aerobic, strength, flexibility and balance exercises [24, 27–29]. Three studies delivered components additional to exercise, which included education on bone health [26], and nutritional [22] and behavioural change interventions [30]. In one study, volunteers conducted home visits and telephone calls to encourage older people to perform home-based exercises [25] but the main exercise intervention was conducted in community classes led by trained personnel.

The frequency of physical activity interventions ranged from weekly (four studies), twice weekly (three studies) to three times a week (one study). Intervention settings included community exercise groups (*n* = 4), home (*n* = 2) and care homes (*n* = 2). The duration of the intervention for the eight studies ranged from 4 weeks to 4 years (Table 1).

### Intervention adherence

Only three studies (the RCTs) reported adherence to the intervention ranging from 30 to 90% depending on adherence definition and duration of the intervention. The recommended number of home visits in the home-based physical and nutritional intervention RCT was 20 visits [22]. Adherence rate was defined as the number of completed visits, which was high at 90% (18 visits, SD 4.6). In the 12 month study by Iliffe et al., volunteers were recruited to support participants in Otago exercise programme arm through home visits and telephone calls. Participants were classed as adherent if they completed 1620 min (75%) of the total 2160 min expected activity over 12 months. 39% of participants in the volunteer arm were classed as adherent and included for analysis. Reasons for non-adherence to the intervention were not provided in both studies. In the 12 month nursing home-based study by Chen et al. [24], 19 (30%) out of the 64 participants completed all of the 72 volunteer-led intervention sessions. The mean number of sessions per participant was 68 (SD 6.1). Common reasons for non-adherence to the intervention included hospitalisation (25.6%), physical discomfort or illness (16.1%) and infection control reasons (13.2%).

### Outcome measures

#### Physical activity levels

The impact of interventions on PA levels was mixed. Two studies measured physical activity levels subjectively using the Community Healthy Activities Model Program for Seniors scale (CHAMPS) and the Physical Activity Scale for the Elderly (PASE) questionnaires. In the three-arm multicentre cluster, RCT conducted by Iliffe et al. [25], participants who were randomised to the Otago exercise programme (OEP) arm received additional support and motivation through home visits and telephone calls by volunteers. The proportion of participants in the OEP who reported reaching the recommended PA target of 150 min of moderate vigorous physical activity (MVPA) calculated from the CHAMPS questionnaire rose from 41 to 43%, compared to a rise of 37.5–38% in the control group, with no statistically significant difference. The home-based physical and nutritional intervention RCT [23] demonstrated an improvement in PA levels measured by the PASE questionnaire among the intervention group, with a mean difference of 9.1 min/day (95% CI 0.9–17.4) spent performing light sport activity. There was also an improvement in overall activity levels in the experiment group by 0.69 h/day (95% CI 0.21–1.2), compared to control group.

#### Functional status

Four studies measured the impact of the volunteer-led intervention on functional outcomes. Measures reported included short physical performance battery (SPPB), timed up and go test (TUG), single leg stand, step touch test, 30 s chair stand test and functional reach.

In the home-based physical and nutritional intervention RCT (*n* = 39, mean age 83 years, SD 8), volunteers delivered twice weekly strength exercises at home, with the use of an elastic resistance band plus nutrition advice. The study found that participants in the intervention arm showed improvement in mean SPPB score by 1.0 (*p* < 0.05) compared to the control group who received social interaction and support.

A quasi-experimental study (Nanduri et al.) among older adults living at home (*n* = 25, mean age 74.4 years, SD 8.5) and at assisted living accommodation (*n* = 17, mean age 86.6 years, SD 5.7) explored the impact of a volunteer-led exercise focusing on posture, balance, strength and flexibility on functional outcomes at 6 months (26). The weekly exercise classes were held at the assisted living facility. The study found improvement in several outcome measures including functional reach (4.4 cm, *p* < 0.001), timed up and go (TUG) (2.3 s, *p* < 0.001) and 30 s(s) chair stand (4 s, *p* < 0.001), compared to baseline score, among older adults living at home. Among older adults in assisted living, there was in improvement in TUG score by 1.7 (*p* < 0.05).

The quasi-experimental study by Waters et al. (*n* = 52, mean age 76.5 years, SD 7.4) measured the impact of weekly volunteer-led exercise classes on various functional measures including TUG test, single leg stand, step touch test, 30 s chair stand test, and functional reach. Volunteers delivered weekly exercise classes adapted from the Otago exercise program (strength and balance). The outcomes were compared against a professionally-led Age Concern Otago exercise group (*n* = 52, mean age 77.0 years), and a control group (*n* = 25, mean age 78.4 years) which performed only seated flexibility and range of motion exercises. The study found that at 12 months, participants in the volunteer-led group had better overall scores in chair stand (*p* = 0.01) and step touch test (*p* = 0.001) compared to the control group. There was also no statistically significant difference for any functional measures comparing the volunteer-led group with the Age Concern Otago group (all *p* > 0.05).

A cluster RCT by Chen et al. which included 10 nursing homes (mean age 78.8 years, SD 7.6) found that older adults in wheelchairs [*n* = 64, mean Barthel Index (BI) score 58, SD 24] who received the Wheelchair-bound Senior Elastic Band (WSEB) exercise programme three times a week, continued to maintain their physical function, at 6 months (BI score 54, 95% CI 51–57) and at 12 months [BI score 56, 95% CI (52–59) [24]. This was compared to participants in the control group (*n* = 63, mean BI score 52, SD 24] experienced functional decline at 6 months (BI score 49, 95% CI 46–53) and at 12 months (BI score 46, 95% CI 43–50). Differences in BI score between the intervention and control group at 6 months (5, 95% CI 0.4–9) and 12 months (9, 95% CI 4–14) were both statistically significant.

#### Frailty status

Three studies included frailty or markers of frailty as an outcome measure. The measures included SHARE-FI, grip strength, and the use of long-term care insurance.

The home-based physical and nutritional intervention RCT found no statistically significant difference in mean grip strength (1.3 kg, 95% CI – 0.3 to 2.9) between experimental and control group. However, a sub-group analysis on frailty status found that participants who were classified as frail according to SHARE-FI, had an odds ratio of 2.7 (95% CI 1.0–7.2) in improvement of handgrip by 2 kg, compared to robust or pre-frail participants. The large prospective cohort study by Yamada et al. (*n* = 3240) measured the impact of volunteer-led group exercise classes on the use of long-term care insurance (LTCI) among community-dwelling older adults aged 65 years and older [28]. The use of LTCI is an indicator of frailty or disability. 1620 older adults received twice weekly volunteer-led exercises which consisted of mild intensity aerobic exercises, mild strength training, flexibility and balance exercises. Over a four year follow-up period, 15.2% of the intervention group participants were issued LTCI certifications as compared to 20.6% in the control group (HR 0.73, 95% CI 0.62–0.86). The cluster RCT by Chen et al. [24] demonstrated an improvement in mean difference of hand grip strength by 1.6 kg (95% CI 0.6–2.5) at 6 months and 3.1 kg (95% CI 1.9–4.4) at 12 months, favouring the resistance exercise group.

#### Fear of falls

Two studies measured fear of falls as an outcome measure and both reported reduction in fear of falling. The home-based physical and nutritional intervention RCT [20] demonstrated an improvement in fear of falling scores, as measured by Falls Efficacy Scale-International (FES-I), with a statistically significant reduction (*p* = 0.016) in FES-I score from 44.1 (SD 13.07) to 39.9 (SD 13.23) in the intervention group, with no change in the control group. A non-randomised single group repeated measure design study (*n* = 335, mean age 78.7 years, SD 8.3) by Healy et al. [30] examined the effectiveness of volunteer-led exercise on fear of falling. The exercise included home-based strength and balance exercise plus cognitive-behavioural techniques to reduce fear of falling. The study found that those who received the intervention had an improvement in fear of falling as measured by the modified FES scale, with a mean change score of 0.195 (*p* < 0.001) at 6 months and 0.205 (*p* = 0.001) at 12 months.

#### Other outcome measures

Two studies compared the use of volunteers- and a professional or healthcare professional-delivered intervention. Izutsu et al. [29] conducted a non-randomised intervention study to compare the effectiveness of an exercise intervention implemented by trained volunteers (*n* = 50, mean age 75.6 years) and health professionals (*n* = 55, mean age 78.3 years) on health-related quality of life measured by SF-36 among community-dwelling older women in Japan. The study found that both interventions maintained or improved all quality of life components in the SF-36 in > 70% of participants, apart from bodily pain. In Waters et al.’s quasi-experimental study comparing the effectiveness of peer-led exercise classes with Age Concern Otago group exercise and controls. All functional measures (chair stand, step touch right, timed up and go test, and functional reach) were improved in both the peer-led and Age Concern Otago group at 12 months, compared to control (*p* < 0.02) but with no statistically significant difference between intervention groups.

#### Adverse events

All studies reported on adverse events and no serious adverse events occurred. One participant in the home-based physical and nutritional intervention RCT study reported back pain that may have been associated with the exercise programme [20]. Non-serious adverse events reported by the Iliffe et al. study were largely musculoskeletal in nature and included back pain, knee pain, hip pain, plantar fasciitis, worsening sciatica and pulled muscle.

## Discussion

The aim of this review was to explore the impact of trained volunteers on the health outcomes of community-dwelling older adults. The eight studies included in this review were heterogeneous in the interventions delivered, with varied frequency and duration, multimodal intervention and outcomes reported. The sample characteristics including mean age, study settings and functional status also differed between studies. The minimum length of intervention was 12 weeks and the most common frequency of intervention was once weekly (four studies).

The recruitment, training and retention of volunteers were poorly described in all eight studies. Four studies provided minimal details on where the volunteers were recruited from but did not describe the recruitment process. Iliffe et al., were not able to recruit the number of volunteers to target, with a high dropout rate among those who expressed interested (*n* = 209), with 71 volunteers receiving training, and 38 volunteering. Several factors which impacted on volunteer disengagement included the length of time between training and beginning work, and the distance volunteers would need to travel to support participants [25]. On the use of volunteers to support or deliver exercise programmes, Iliffe et al. concluded that feasibility studies on the use of trained volunteers should be conducted prior to embarking on a large scale trial. Volunteers require training and good support mechanisms to ensure that they carry out their role well with help provided when needed. This require investment in labour and time and thus the role of the coordinator who is responsible for recruitment and training is very important [16].

The best evidence on the impact of volunteer-led intervention on physical activity and health outcomes were from two RCTs. The RCT examining the impact of a twice weekly volunteer-led home-based strength exercise and nutrition advice intervention in pre-frail and frail (defined by SHARE-FI) community-dwelling older adults showed improvement in physical activity levels [23], grip strength [19], and nutritional status [22]. The study also showed reduction in frailty scores [22] and fear of falling [20]. The second RCT by Chen et al. showed that a thrice-weekly volunteer-led resistance exercise training using elastic bands among wheelchair-bound nursing home adults resulted in improvement in grip strength and functional status [24].

This review found some evidence on the impact of volunteer-led physical activity intervention on physical activity levels of older people, although the findings were mixed. Iliffe et al. found that the proportion of participants in the volunteer-led arm who reported spending at least 150 min of MVPA rose from 41 to 43% but this was not statistically significant. The home-based physical and nutritional intervention RCT reported that participants who received the volunteer-led intervention showed a statistically significant improvement in overall activity levels 0.69 h/day (95% CI 0.21–1.18). Reduction in primary and secondary prevention of chronic disease [31], improved bone health [32], and improved well-being and quality of life [33] are some of the well-known benefits of increased physical activity. It is important that older adults are encouraged and given the opportunity to be physically active. Waters et al. reported that the psychosocial benefits of having peer-mentors is a motivating factor in exercise adherence [27].

Weaker evidence from non-randomised experimental studies showed that (i) twice weekly home-based strength and balance exercises plus a cognitive and behavioural intervention among older adults at risk of falls resulted in a reduction in fear of falling [30]; and (ii) weekly group exercise which include posture, balance, strength and flexibility plus additional education on bone health among older adults in assisted living resulted in improvement in physical function. A common element in these studies is the inclusion of strength or resistance exercise training. Participants in these studies were also frail, functionally impaired, or at risk of falls. These findings agree with a systematic review which was conducted by Lopez et al. to explore the benefits of resistance training in physically frail older adults [34]. The review which included 16 studies found that older adults who were classified as frail using a valid frailty criteria, or those who were functionally impaired or institutionalised, benefited from resistance exercise training alone or a multimodal training, with improvement in muscle mass (3.4–7.5%) and strength (6.6–37%), and in functional capacity (4.7–58.1%). There is convincing evidence that healthy older adults benefit from resistance training [35, 36] and evidence suggest that this benefit extends even to frail older adults [37].

Grip strength is a recommended simple measure of muscle strength in the diagnosis of sarcopenia [38], and a marker of frailty in older adults [39]. The review found evidence of improvement in grip strength through volunteer-led physical activity interventions. Chen et al. showed an improvement in grip strength by 3.1 kg (95% CI 1.9–4.4) in the group-based resistance exercise intervention arm at 12 months, compared to controls. The home-based physical and nutritional intervention RCT found that among frail older adults, the intervention led to an improvement in grip strength, with an odds ratio of 2.7 (95% CI 1.0–7.2) in improvement of handgrip by 2 kg, compared to robust or pre-frail older adults. This finding is consistent with existing literature which suggests that among older adults, particularly the oldest-old, frail females, or those living in long-term care facilities, tend to benefit the most from exercise interventions [40]. A recently published meta-analysis which included 24 trials (*n* = 3018, mean age 73.3 years) demonstrated a small but statistically significant difference in standardised mean difference in grip strength (0.28, 95% CI 0.13–0.44) among healthy community-dwelling older adults who received resistance training and multimodal intervention training [41]. Yamada et al. were also able to demonstrate improvement in frailty and disability levels at 4 years, as indicated by the use of long-term care insurance, among Japanese older adults who received once or twice weekly volunteer-led group exercise which included aerobic, strength and balance exercises. Evidence from the studies included in this review suggests that volunteers can be trained to deliver physical activity interventions, with evidence of improved frailty status.

Falls in older adults are the major cause of injury-related hospitalisation among those aged > 65 years [42]. Falls increase the fear of falling among older adults, which in turn, increases their risk of falling through a reduction in daily physical activity, loss of self-confidence, and change in gait parameters [43]. Fear of falls is an important outcome measure to consider in older adults as it can lead to functional decline [44], restriction of social participation [45, 46] and decreased quality of life [47]. Findings from the Kapan et al. study [20] suggest that home-based strength exercise plus nutritional intervention could improve fear of falling, as measured by Falls Efficacy Scale, among older adults. The non-randomised single group study by Healy et al. [30] also demonstrated a reduction in fear of falling at 12 months among community-dwelling older adults who received twice weekly volunteer-led strength and balance exercise.

The adherence rates reported by the three RCTs were variable, with two trials (Chen [24] and Iliffe [25]) reporting low adherence, and the home-based physical and nutritional intervention RCT (Luger et al.) reporting higher adherence. This is likely to be related to the duration of the study as both the Chen et al. and Iliffe et al. studies had a 12 months intervention period compared to Luger et al., which had a 12 weeks intervention period.

### Limitations of the study

This review has some limitations. Non-English studies were excluded, which could potentially introduce language bias and limit the generalisability of the findings from this review. Five articles in this review reported findings from the same study, which could potentially introduce multiple publication bias. However, meta-analysis was not conducted due to study heterogeneity and thus duplication of data was not of concern in this review. Only three RCTs were identified for this review, and two of which were small trials. Non-randomised experimental studies were also included in this review. Although definitive conclusions on the cause and effect of the volunteer-led interventions cannot be drawn from these studies, findings from these studies agreed largely with the outcomes from the RCTs, including improvement in functional outcomes, and reduction in fear of falls. A grey literature search was not conducted where sources such as service improvement projects may have been identified, which may have relevance particularly in the field of volunteering.

## Conclusion

This review reports eight studies including three RCTs which shows some evidence that volunteer-led physical activity interventions that include resistance exercise training, may improve outcomes of community-dwelling older adults including improved functional status, frailty status, and reduction in fear of falls. More high-quality RCTs are needed to investigate the effects of volunteer-led PA interventions among older people. All interventions had a minimum duration of 12 weeks and the most common frequency was once weekly intervention. Volunteer-led PA interventions are safe, with no serious adverse events reported in trials. Feasibility studies using a mixed method approach are needed to explore the practicalities of volunteer recruitment and training, identify barriers and facilitators of recruiting, training and retaining volunteers to deliver physical activity interventions.

## Data Availability

The datasets used and/or analysed during the current study are available from the corresponding author on reasonable request.

## References

[1] Stevens Z, Barlow C, Kendrick D (2014). Effectiveness of general practice-based physical activity promotion for older adults: systematic review. Prim Health Care Res Dev.

[2] Chase JAD, Phillips LJ, Brown M (2017). Physical activity intervention effects on physical function among community-dwelling older adults: a systematic review and meta-analysis. J Aging Phys Act.

[3] 2008 Physical activity guidelines for Americans: Be active, healthy and happy! Rockville: U.S. Department of Health and Human Services; 2008. Available from: https://health.gov/sites/default/files/2019-09/paguide.pdf. Accessed 21 Apr 2020

[4] Foster C, Reilly J, Jago R et al. (2019) UK Chief Medical Officers' Physical Activity Guidelines. GOV.UK: UK Department of Health and Social Care 2019. Available from: https://assets.publishing.service.gov.uk/government/uploads/system/uploads/attachment_data/file/832868/uk-chief-medical-officers-physical-activity-guidelines.pdf. Accessed 21 Apr 2020

[5] World Health Organization (2010) Global recommendations on physical activity for health 2010 [28/07/2015]. Available from: https://www.who.int/dietphysicalactivity/factsheet_olderadults/en. Accessed 21 Apr 2020 26180873

[6] Sparling PB, Howard BJ, Dunstan DW (2015). Recommendations for physical activity in older adults. BMJ.

[7] Harris TJ, Owen CG, Victor CR (2009). What factors are associated with physical activity in older people, assessed objectively by accelerometry?. Br J Sports Med.

[8] Older Americans (2010) Key indicators of well-being. Federal interagency forum on aging-related statistics; 2012. Available from: https://agingstats.gov/docs/PastReports/2010/OA2010.pdf. Accessed 21 Apr 2020

[9] Stevens G (2009). Global health risks: progress and challenges. Bull World Health Organ.

[10] British Heart Foundation. Physical activity statistics (2015). https://www.bhf.org.uk/informationsupport/publications/statistics/physical-activity-statistics-2015. Accessed 21 Apr 2020

[11] Lee IM, Shiroma EJ, Lobelo F (2012). Effect of physical inactivity on major non-communicable diseases worldwide: an analysis of burden of disease and life expectancy. Lancet.

[12] da Silva VD, Tribess S, Meneguci J (2019). Association between frailty and the combination of physical activity level and sedentary behavior in older adults. BMC Public Health.

[13] Naylor C, Mundle C, Weaks L et al (2013) Volunteering in health and care: Securing a sustainable future 2013. Available from: https://www.kingsfund.org.uk/publications/volunteering-health-and-care. Accessed 21 Apr 2020

[14] Galea A, Naylor C, Buck D (2013). Volunteering in acute trusts in England: understanding the scale and impact.

[15] Stewart AL, Gillis D, Grossman M (2006). Diffusing a research-based physical activity promotion program for seniors into diverse communities: CHAMPS III. Prev Chronic Dis.

[16] Peel NM, Warburton J (2009). Using senior volunteers as peer educators: what is the evidence of effectiveness in falls prevention?. Australas J Ageing.

[17] Moher D, Liberati A, Tetzlaff J (2009). Preferred reporting items for systematic reviews and meta-analyses: the PRISMA statement. BMJ.

[18] Nuotio MS, Luukkaala T, Tammela T (2019). Elevated post-void residual volume in a geriatric post-hip fracture assessment in women-associated factors and risk of mortality. Aging Clin Exp Res.

[19] Haider S, Dorner TE, Luger E (2017). Impact of a home-based physical and nutritional intervention program conducted by lay-volunteers on handgrip strength in prefrail and frail older adults: a randomized control trial. PLoS ONE.

[20] Kapan A, Luger E, Haider S (2017). Fear of falling reduced by a lay led home-based program in frail community-dwelling older adults: a randomised controlled trial. Arch Gerontol Geriatr.

[21] Kapan A, Winzer E, Haider S (2017). Impact of a lay-led home-based intervention programme on quality of life in community-dwelling pre-frail and frail older adults: a randomized controlled trial. BMC Geriatr.

[22] Luger E, Dorner TE, Haider S (2016). Effects of a home-based and volunteer-administered physical training, nutritional, and social support program on malnutrition and frailty in older persons: a randomized controlled trial. J Am Med Dir Assoc.

[23] Winzer E, Dorner TE, Grabovac I (2019). Behavior changes by a buddy-style intervention including physical training, and nutritional and social support. Geriatr Gerontol Int.

[24] Chen KM, Li CH, Huang HT (2016). Feasible modalities and long-term effects of elastic band exercises in nursing home older adults in wheelchairs: a cluster randomized controlled trial. Int J Nurs Stud.

[25] Iliffe S, Kendrick D, Morris R (2014). Multicentre cluster randomised trial comparing a community group exercise programme and home-based exercise with usual care for people aged 65 years and over in primary care. Health Technol Assess.

[26] Nanduri AP, Fullman S, Morell L (2018). Pilot study for implementing an osteoporosis education and exercise program in an assisted living facility and senior community. J Appl Gerontol.

[27] Waters DL, Hale LA, Robertson L (2011). Evaluation of a peer-led falls prevention program for older adults. Arch Phys Med Rehabil.

[28] Yamada M, Arai H (2017). Self-management group exercise extends healthy life expectancy in frail community-dwelling older adults. Int J Environ Res Public Health.

[29] Izutsu K, Arima K, Abe Y (2017). Exercise intervention implemented by trained volunteers improves health-related quality of life among Japanese community-dwelling older females: an intervention study. J Phys Ther Sci.

[30] Healy TC, Peng C, Haynes MS (2008). The feasibility and effectiveness of translating a matter of balance into a volunteer lay leader model. J Appl Gerontol.

[31] Warburton DER, Nicol CW, Bredin SSD (2006). Health benefits of physical activity: the evidence. CMAJ Can Med Assoc J.

[32] Liu-Ambrose TY, Khan KM, Eng JJ (2004). Both resistance and agility training increase cortical bone density in 75- to 85-year-old women with low bone mass: a 6-month randomized controlled trial. J Clin Densitom.

[33] Fox KR, Stathi A, McKenna J (2007). Physical activity and mental well-being in older people participating in the Better Ageing Project. Eur J Appl Physiol.

[34] Lopez P, Pinto RS, Radaelli R (2018). Benefits of resistance training in physically frail elderly: a systematic review. Aging Clin Exp Res.

[35] Vikberg S, Sörlén N, Brandén L (2019). Effects of resistance training on functional strength and muscle mass in 70-year-old individuals with pre-sarcopenia: a randomized controlled trial. J Am Med Dir Assoc.

[36] Jadczak AD, Makwana N, Luscombe-Marsh N (2018). Effectiveness of exercise interventions on physical function in community-dwelling frail older people: an umbrella review of systematic reviews. JBI Database Syst Rev Implement Rep.

[37] Kidd T, Mold F, Jones C (2019). What are the most effective interventions to improve physical performance in pre-frail and frail adults? A systematic review of randomised control trials. BMC Geriatr.

[38] Cruz-Jentoft AJ, Bahat G, Bauer J (2019). Sarcopenia: revised European consensus on definition and diagnosis. Age Ageing.

[39] Fried LP, Tangen CM, Walston J (2001). Frailty in older adults: evidence for a phenotype. J Gerontol A Biol Sci Med Sci.

[40] Theou O, Stathokostas L, Roland KP (2011). The effectiveness of exercise interventions for the management of frailty: a systematic review. J Aging Res.

[41] Labott BK, Bucht H, Morat M (2019). Effects of exercise training on handgrip strength in older adults: a meta-analytical review. Gerontology.

[42] Gale CR, Cooper C, Aihie SA (2016). prevalence and risk factors for falls in older men and women: the English Longitudinal Study of Ageing. Age Ageing.

[43] Friedman SM, Munoz B, West SK (2002). Falls and fear of falling: which comes first? A longitudinal prediction model suggests strategies for primary and secondary prevention. J Am Geriatr Soc.

[44] Cumming RG, Salkeld G, Thomas M (2000). Prospective study of the impact of fear of falling on activities of daily living, SF-36 scores, and nursing home admission. J Gerontol A Biol Sci Med Sci.

[45] Howland J, Peterson EW, Levin WC (1993). Fear of falling among the community-dwelling elderly. J Aging Health.

[46] Zijlstra GAR, van Haastregt JCM, Eijk JTM (2007). Prevalence and correlates of fear of falling, and associated avoidance of activity in the general population of community-living older people. Age Ageing.

[47] Arfken CL, Lach HW, Birge SJ (1994). The prevalence and correlates of fear of falling in elderly persons living in the community. Am J Public Health.

